# Robot‐assisted Percutaneous Radiofrequency Ablation for the Treatment of Osteoid Osteomas

**DOI:** 10.1111/os.14043

**Published:** 2024-03-31

**Authors:** Ka Li, Jianmin Li, Zonghao Li, Zhiping Yang, Xin Li, Qiang Yang, Yuantong Liu, Zhenfeng Li

**Affiliations:** ^1^ Department of Orthopedics Qilu Hospital of Shandong University Jinan China

**Keywords:** Osteoid osteoma, Radiofrequency ablation, Surgical robot

## Abstract

**Objective:**

Percutaneous CT‐guided radiofrequency ablation (CT‐RFA) is a widely accepted procedure for treatment of osteoid osteomas. However, the application of CT‐RFA was restricted as a result of some drawbacks, such as radiation exposure, and inconvenience in general anesthesia. The primary aim of this study is to evaluate the safety and efficacy of intra‐operative TiRobot‐assisted percutaneous RFA of osteoid osteomas.

**Methods:**

We retrospectively reviewed 21 medical files of patients who were treated with percutaneous RFA of osteoid osteomas guided by the TiRobot system in our institution between March 2021 and April 2022. The three‐dimensional images obtained by a 3D C‐arm intra‐operatively were sent to the TiRobot system. The puncture point and trajectory were designed. Then the guide pin was positioned to the lesion with the assistance of TiRobot and the biopsy sheath was inserted into the lesion through the guide pin. The tumor was biopsied for pathological examination. Then the RFA needle was inserted into the nidus through the biopsy sheath for thermal ablation. Data were extracted on the associated complications, the reduction in pain at 1 month and 1 year postoperatively assessed by the visual analogue scale (VAS). A paired *t*‐test was used to compare the pre‐operative and post‐operative VAS scores.

**Results:**

The patients included 17 males and four females with a mean age of 19.5 ± 10.4 years (range 3–45 years). Lesions were located on the femur in nine cases, on the tibia in nine cases, on the humerus in one case, on the calcaneus in one case, and on the acetabulum in one case. TiRobot‐assisted percutaneous RFA was successfully performed on all 21 patients. There was no intra‐operative or post‐operative complications observed. Pathological diagnosis of osteoid osteoma was obtained in 11 patients, but the other 10 cases were not pathologically diagnosed. The mean follow‐up time was 18.8 months (range: 12–26 months).Post‐operative VAS scores were reduced significantly in all cases. The mean VAS score decreased from 6.5 pre‐operatively to 0.5 at 1 month post‐operatively and to 0.1 at 1 year post‐operatively.

**Conclusion:**

As a reliable technique for localizing and resection of nidus, TiRobot‐assisted percutaneous RFA is a safe and effective option for the treatment of osteoid osteomas.

## Graphical abstract

Intra‐operative TiRobot‐assisted percutaneous radiofrequency ablation (RFA) of osteoid osteomas is a reliable technique for localizing and ablation of nidus. The procedure is a safe and effective option for the treatment of osteoid osteomas.

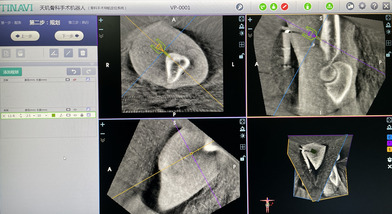

## Introduction

Osteoid osteoma is a benign osteoblastic bone tumor that most commonly occurs in the extremities of children and young adults with a male‐to‐female ratio of 3: 1. Osteoid osteomas account for approximately 10% of all benign bone tumors. The most common site is meta‐diaphyseal location of femur and tibia. The tumor characteristically presents with nocturnal pain at the site of involvement which may be relieved by non‐steroidal anti‐inflammatory drugs (NSAIDs).

Osteoid osteoma is reflected on radiography and CT as a solitary radiolucent nidus with variable degree of surrounding sclerosis. In terms of treatment, excision or destruction of the nidus has been proven to be curative. Traditionally, open surgical resection for osteoid osteomas was effective. However, this might result in over‐excision of bone and inaccurate localization of the nidus, which led to instability, pathological fracture, long recovery time and tumor recurrence.^1^, ^2^ Various minimally invasive techniques consisted of percutaneous core drilling, percutaneous radiofrequency ablation (RFA) and laser photocoagulation, have been described.^3^, ^4^, ^5^ Percutaneous CT‐guided radiofrequency ablation (CT‐RFA) with high clinical success, brief recovery and low complication rate, has replaced surgical resection as the reference treatment for osteoid osteomas. The safety and efficacy of this procedure has been supported by a large number of studies.

However, repeated CT scans and multiple times of angle adjustment are necessary during the procedure to guide the needle to the nidus in a controlled fashion, which most likely increases the operating time together with the radiation exposure to patients. In addition, the pain is quite severe when the puncture needle is inserted into the nidus during the operation. Most patients of osteoid osteomas are children who need to accept general anesthesia in the operating room. A CT scan may not be undertaken in operating rooms in some parts of hospitals. So CT‐guided RFA in the operating room for child patients sometimes cannot be conducted in clinical work. The application of CT‐RFA was restricted as a result of these drawbacks. The problem needs to be resolved so that reliable image‐assisted percutaneous RFA of osteoid osteomas is conducted with satisfactory anesthetic effect in the operating room. To overcome these drawbacks, several studies have recently revealed that using three‐dimensional intra‐operative image in combination with real‐time navigation allows the surgeon to find the safest and most accurate tract to the nidus of osteoid osteomas.^6^, ^7^, ^8^ In addition, intra‐operative 3D images in combination with surgical robots are able to accurately direct implants into demanding anatomical locations with maximum accuracy and minimum ionizing radiation.^9^, ^10^, ^11^ This prompts us to introduce this method into the treatment of osteoid osteoma combined with RFA.

The aim of this study is to introduce a new method that utilizes a robot system combined with a 3D C‐arm to assist RFA in osteoid osteoma and assess the safety and efficacy of this procedure.

## Methods

### Patient Selection

This study retrospectively analyses patients with osteoid osteoma who underwent treatment with TiRobot‐assisted percutaneous RFA between March 2021 and April 2022 in the Department of Orthopedic Oncology of Qilu hospital, Shandong University, China. The procedure was approved by the Institutional Review Board and Ethics Committee of Qilu Hospital of Shandong University (KYLL‐2022(ZM)‐585).

**FIGURE 1:**
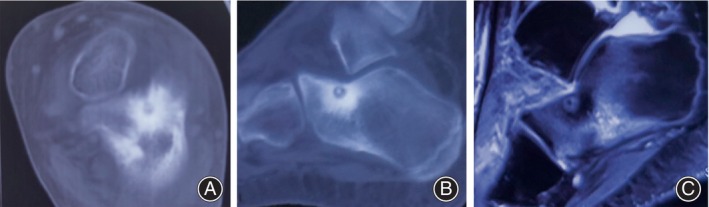
Pre‐operative images of osteoid osteoma in the calcaneus in a 11‐year‐old male. (A, B) Axial and sagittal images of CT scan showed an radiolucent nidus with extensive surrounding sclerosis (C) Sagittal T2‐weighted‐fat supress (T2W‐FS) sequence of MRI. showed the perilesional bone oedema.

The inclusion criteria were: (i) patients with complete basic information, laboratory examination and imaging examination; (ii) patients with osteoid osteomas confirmed by clinical finding, X‐ray, CT scan and magnetic resonance imaging (MRI) (Figure 1); (iii) patients who presented with local pain; (iv) patients treated with TiRobot‐assisted percutaneous RFA; and (v) patients who did not receive prior NSAIDs treatment 1 week pre‐operatively and post‐operatively or prior surgical treatment. The exclusion criteria were: (i) patients unable to complete follow‐up; (ii) patients with systemic or local infection; and (iii) patients with blood coagulation dysfunction, and cardiopulmonary, liver, and renal failure. A total of 21 patients were involved in this study.

### Surgical Procedure

**FIGURE 2:**
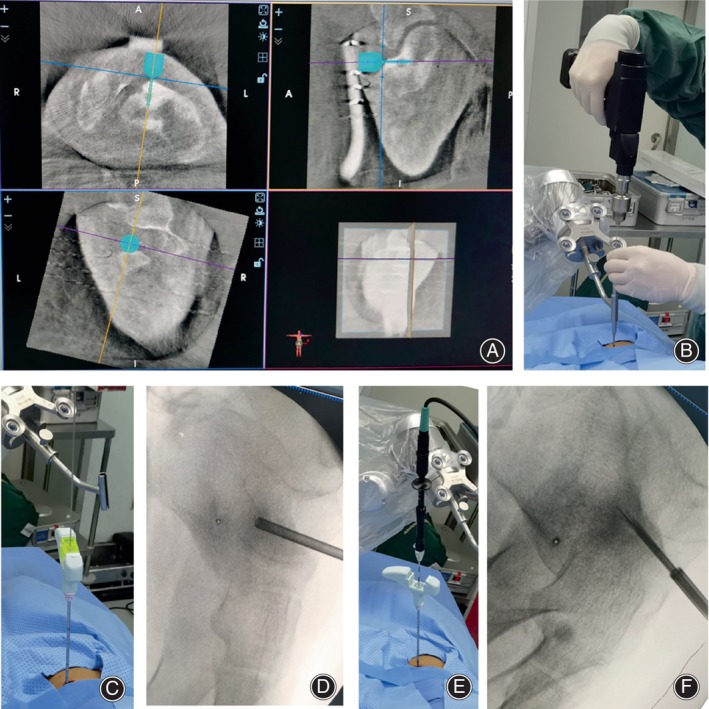
An 11‐year‐old patient with osteoid osteoma in the calcaneus treated with percutaneous radiofrequency ablation (RFA) with the assistance of TiRobot. (A) The three‐dimensional images was sent to TiRobot system and the puncture point and trajectory were designed in the robot system. (B) The guide pin was inserted to the lesion with the assistance of robotic arm according to the planed path. (C, D) The biopsy sheath was inserted into the nidus through guide pin for pathological examination and the location was confirmed by a scan. (E, F) The RFA needle was inserted into the nidus through biopsy sheath, which was confirmed by a repeat scan, followed by ablation according to a protocol of 90°C for 5 min.

The surgical procedure was carried out in the operating room. After general anesthesia, the patient was placed on a radiolucent operating table. The affected extremity was prepared and draped in a sterile manner. The reference tracker was secured to the patient by anchor devices installed on the operating table. The patient was then scanned by a 3D C‐arm (Siemens Healthineers, Marburg, Germany) to locate the lesion. The three‐dimensional images was created and sent to TiRobot system (TINAVI Medical Technologies, Beijing, China). The puncture point and trajectory were designed. The robotic arm was adjusted according to the selected trajectory. A catheter was attached at the end of the robotic arm, allowing surgeons to implant a guide pin. Then the guide pin was inserted to the lesion with the assistance of a robotic arm and the biopsy sheath was inserted into the lesion through the guide pin. The tumor was biopsied for pathological examination. Then the RFA needle (RITA Angiodynamics, Latham, NY, USA) was inserted into the nidus through the biopsy sheath. After ensuring correct placement of the RFA needle using the 3D C‐arm, an RFA was carried out at 90°C for 5 min (Figure 2). The RFA needle and biopsy sheath were removed. The incision was examined for burns or other superficial complications and was dressed up with aseptic bandages.

The visual analog scale (VAS) was applied to assess the pain before and after the procedure. Radiological examination was performed to observe the changes of the lesion.

### Statistical Analysis

SPSS 21.0 was used for statistical analysis (IBM, Armonk, NY, USA). A paired *t*‐test was used to compare the pre‐operative and post‐operative VAS scores. *P*‐values < 0.05 were considered as statistically significant.

## Results

### General Results

The patients included 17 males and four females with a mean age of 19.5 ± 10.4 years (range 3–45 years). The lesions were located on the femur in nine cases, on the tibia in nine cases, on the humerus in one case, on the calcaneus in one case, and on the acetabulum in one case (Table S1). TiRobot‐assisted percutaneous RFA was successfully performed on all 21 patients. The operation time, from locating tumor to completing RFA, was 38.71 ± 7.53 min. There was no complication associated with the procedure observed.

### Functional Results

The mean follow‐up time was 18.8 months (range: 12–26 months). There was no pain recurrence and post‐operative VAS scores were reduced significantly in all cases. The mean VAS score decreased from 6.5 pre‐operatively to 0.5 at 1 month post‐operatively and to 0.1 at 1 year post‐operatively (*p* < 0.05).

### Pathological Results

Pathological diagnosis of osteoid osteoma was obtained in 11 patients. There was no adequate biopsy specimen in the remaining 10 cases for pathological diagnosis.

## Discussion

This study demonstrated that TiRobot‐assisted percutaneous RFA is a feasible treatment of osteoid osteomas. Moreover, no intra‐operative or post‐operative complication occurred, and the pain of patients was significantly relieved. This method is a safe and effective option for the treatment of osteoid osteomas.

### CT‐Guided RFA and Improvement

CT‐guided RFA is considered as the mainstay for treatment of osteoid osteomas. This minimally invasive procedure provides excellent clinical results with rare complications and lowers hospital stay. CT scan is the golden standard for locating the nidus and for guiding treatments. However, CT scans have drawbacks including radiation exposure due to multiple adjustment during the operation, inflexibility that general anesthesia and CT scan may not be obtained simultaneously.

On this basis, some improvement has been introduced in terms of guidance during operation. Yu *et al*. reported 3D Iso‐C C‐arm navigation‐guided RFA as a safe and effective option with real‐time imaging of the anatomy, accurate resection of the tumor and convenient confirmation of the procedure results.^6^ Ankory *et al*. also found that the O‐arm and stealth navigation lowered radiation exposure compared with the traditional CT‐guided technique.^7^ In addition, adding laser guidance to CBCT‐guided osteoid osteoma RFA significantly reduced fluoroscopy time without increasing procedure time compared to freehand CBCT guidance.^12^

### Feasibility and Effectiveness of TiRobot‐assisted Percutaneous RFA

**FIGURE 3:**
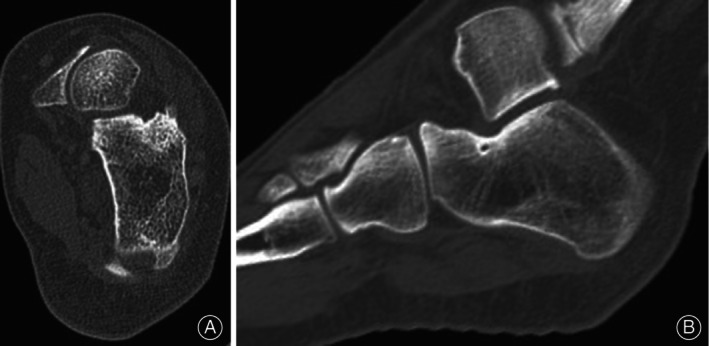
Post‐operative CT images of osteoid osteoma in the calcaneus in an 11‐year‐old male. Post‐operative axial (A) and sagittal (B) images of CT at 12th month showed that the nidus shrank and achieved significant bone remolding.

**FIGURE 4:**
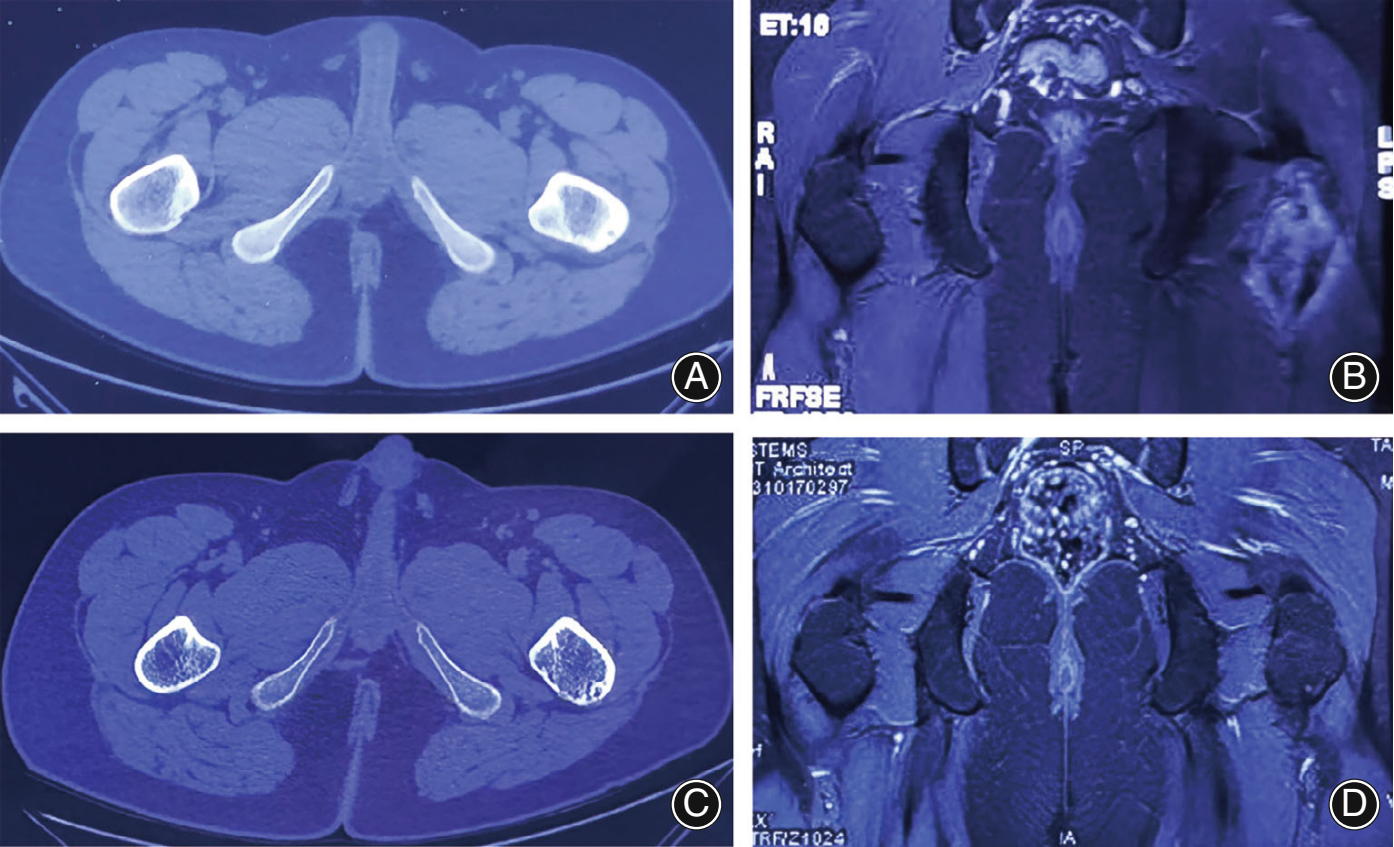
Pre‐operative and post‐operative images of a 14‐year‐old male confirmed with osteoid osteoma in the left femur using TiRobot‐assisted percutaneous RFA. (A) Pre‐operative axial CT image. (B) Pre‐operative coronal MR image. (C) Post‐operative axial CT image at 12th month showed bone remolding (D) Post‐operative coronal MR image at 12th month showed resolution of bone oedema around the nidus.

In our study, 3D imaging combined with The iRobot were introduced for guidance in osteoid osteoma RFA. The procedure was successfully performed on all 21 patients without peri‐operative complications. The reduction of pain was significant. Osteoid osteomas are characteristic for localized pain, but the technique provides precise locating and guidance, and can be considered as effective in the term of clinical pain relief. In addition, the effective ablation can be demonstrated by some imaging findings including nidus shrinking, resolution of bone oedema and the presence of bone remodeling in the follow‐up (Figures 3 and 4).

3D imaging combined with robotic arm allow surgeons to find an optimum entry point and tract to the nidus instead of manual procedure, which ensures the accuracy and efficacy of surgery. In terms of radiation exposure, 3D C‐arm scan is only performed twice during the whole procedure, for 3D images collection before registration and confirming the position of RFA needle, mitigates relatively high doses of radiation of CT‐guided RFA. It has been demonstrated that robot‐assisted RFA with three‐dimensional imaging system is a precise procedure with less radiation exposure compared with the conventional CT‐guided radiofrequency ablation in the treatment of osteoid osteoma (436.25 ± 327.66 mGy‐cm^2^ *vs* 776.05 ± 474.58 mGy‐cm^2^, *p* < 0.05).^13^ In addition, The operation time was shorter in the robot‐assisted RFA group than CT‐guided RFA group (40.29 ± 9.05 min *vs* 58.18 ± 12.47 min, *p* < 0.05).^13^ Another advantage of the robot‐assisted RFA is that the procedure is carried out in the operating room, allowing general anesthesia and transfer to open surgery when required. It improves patients compliance during operation, especially for children and adolescents. For less invasiveness, the tracer is fixed *via* anchor devices installed on the operating table, which avoids drilling K‐wires into the involved bone. Only one skin incision with a length of about 3 mm is needed.

Despite the benefits above, this procedure requires skilled personnel and an expensive setup, which may not be available in some hospitals. At the same time, definite pathological diagnosis was obtained in 11 out of the 21 patients. The other 10 patients had inadequate biopsy specimens for pathological diagnosis. Cantwell *et al*. reports that histological confirmation with minimal‐access techniques is only available in approximately 36–75% of cases.^14^ However, pathological diagnosis is important especially for untypical clinical or radiological manifestation. Pre‐operative biopsy or conventional method that provides adequate pathology samples should be selected in these patients.

### Surgical Tip

The surgical procedure is carried out in the operating room under general anesthesia. The reference tracker is secured to the patient by anchor devices installed on operating table. The three‐dimensional images is created by 3D C‐arm and uploaded to the TiRobot system. The puncture point and trajectory is designed and a guide pin is inserted into the nidus with the assistance of robotic arm. The tumor is biopsied for pathological examination and the RFA needle is inserted into the nidus through biopsy sheath, after which the RFA is performed.

### Limitations and Prospect of Clinical Application

There are several limitations in this study. First, it is a retrospective analysis with a small sample size. Additional clinical studies with a large group of patients and a control group are necessary to further verify the results mentioned in this study. Second, the follow‐up time is short since the technique is newly used in the treatment of osteoid osteomas. In future studies, we will prolong the follow‐up time for a more convincing long‐term results.

With more applications of surgical robots in Orthopedic surgey, intra‐operative 3D images in combination with surgical robots have greatly improved the accuracy of surgery. The procedure introduced in this study may be applied in locating small bone tumors or special anatomic location. It will reduce surgery invasion and radiation exposure to surgeon and patient.

## Conclusion

It is found that RFA guided by intra‐operative 3D imaging in combination with the TiRobot system is a safe and effective technique in the treatment of osteoid osteomas. It may be considered as an alternative to traditional CT‐guided interventions, especially among younger patients. Prospective comparative studies with more patients and longer follow‐up are required to further understand the therapeutic effect and complication rates of this technique.

## Conflict of Interest Statement

Each author declares that neither he, nor any member of his immediate family, has funding or commercial associations (consultancies, stock ownership, equity interest, patent/licensing arrangements, etc.) that might pose a conflict of interest in connection with the submitted article.

## Ethics Statement

The study protocol was approved by the Institutional Review Board and Ethics Committee of Qilu Hospital of Shandong University.

## Author Contributions

All authors had full access to the data in the study and take responsibility for the integrity of the data and the accuracy of the data analysis. Conceptualization, Jianmin Li and Zhenfeng Li; methodology, Zhenfeng Li and Qiang Yang; investigation, Ka Li, Qiang Yang, Zhenfeng Li and Yuantong Liu; formal analysis, Ka Li; resources, Jianmin Li, Zhiping Yang, Xin Li and Qiang Yang; writing—original draft, Ka Li; writing—review and editing, Ka Li and Zhenfeng Li; visualization, Ka Li; supervision, Zhenfeng Li; funding acquisition, Ka Li.

## Authorship Declaration

We declare that that all authors listed meet the authorship criteria according to the latest guidelines of the International Committee of Medical Journal Editors, and that all authors are in agreement with the manuscript.

## Supporting information


**Table S1.** Patient demographic data (n = 21).
