# Supplementary material for: Robot‐assisted Percutaneous Radiofrequency Ablation for the Treatment of Osteoid Osteomas
Source: Orthop Surg. 2024 Mar 31;16(5):1246–51. doi: 10.1111/os.14043 (PMC11062880; doi:10.1111/os.14043)
Supplement: Supplementary file 1 — Table S1. Patient demographic data (n = 21). [file OS-16-1246-s001.docx]

Table 1 Patient demographic data (n = 21)

| Parameter | Values |
| --- | --- |
| Gender |  |
| male | 17 |
| female | 4 |
| Mean age(years) | 19.5(range 3-45 ) |
| Lesion location |  |
| femur | 9 |
| tibia | 9 |
| humerus | 1 |
| calcaneus | 1 |
| acetabulum | 1 |
